# Cell senescence is an antiviral defense mechanism

**DOI:** 10.1038/srep37007

**Published:** 2016-11-16

**Authors:** Maite Baz-Martínez, Sabela Da Silva-Álvarez, Estefanía Rodríguez, Jorge Guerra, Ahmed El Motiam, Anxo Vidal, Tomás García-Caballero, Miguel González-Barcia, Laura Sánchez, César Muñoz-Fontela, Manuel Collado, Carmen Rivas

**Affiliations:** 1 Centro de Investigación en Medicina Molecular (CIMUS), Universidade de Santiago de Compostela, Instituto de Investigaciones Sanitarias (IDIS), Santiago de Compostela, E15706, Spain; 2 Instituto de Investigación Sanitaria de Santiago de Compostela (IDIS), Complexo Hospitalario Universitario de Santiago de Compostela (CHUS), Sergas, Santiago de Compostela, E15706, Spain; 3 Heinrich Pette Institute, Leibniz Institute for Experimental Virology, Hamburg, Germany; 4 Departamento de Genética. Facultad de Veterinaria, Universidade de Santiago de Compostela, Campus de Lugo, Lugo, E27002, Spain; 5 Departamento de Fisioloxía and Centro de Investigación en Medicina Molecular (CIMUS), Universidade de Santiago de Compostela, Instituto de Investigaciones Sanitarias (IDIS), Santiago de Compostela, E15782, Spain; 6 Departamento de Ciencias Morfológicas, Facultad de Medicina. USC. Complejo Hospitalario de Santiago (CHUS), Sergas, E15706 Santiago de Compostela, Spain; 7 Departamento de Farmacia, Xerencia de Xestión Integrada de Santiago de Compostela, SERGAS, Travesía Choupana s/n, Santiago de Compostela E15706, Spain; 8 Clinical Pharmacology Group, Health Research Institute of Santiago de Compostela (IDIS-ISCIII), Sergas, Travesía da Choupana s/n, Santiago de Compostela 15706, Spain; 9 German Center for Infection Research (DZIF), Partner site Hamburg, Hamburg, Germany; 10 Department of Molecular and Cellular Biology, Centro Nacional de Biotecnología-CSIC, Darwin 3, Madrid 28049, Spain

## Abstract

Cellular senescence is often considered a protection mechanism triggered by conditions that impose cellular stress. Continuous proliferation, DNA damaging agents or activated oncogenes are well-known activators of cell senescence. Apart from a characteristic stable cell cycle arrest, this response also involves a proinflammatory phenotype known as senescence-associated secretory phenotype (SASP). This, together with the widely known interference with senescence pathways by some oncoviruses, had led to the hypothesis that senescence may also be part of the host cell response to fight virus. Here, we evaluate this hypothesis using vesicular stomatitis virus (VSV) as a model. Our results show that VSV replication is significantly impaired in both primary and tumor senescent cells in comparison with non-senescent cells, and independently of the stimulus used to trigger senescence. Importantly, we also demonstrate a protective effect of senescence against VSV *in vivo*. Finally, our results identify the SASP as the major contributor to the antiviral defense exerted by cell senescence *in vitro*, and points to a role activating and recruiting the immune system to clear out the infection. Thus, our study indicates that cell senescence has also a role as a natural antiviral defense mechanism.

Cellular senescence is a stable state of proliferative arrest, initially described as the result of proliferative exhaustion *in vitro* and thought to represent cellular aging^1^. The cellular senescence program can be activated by a variety of cell-intrinsic and -extrinsic stresses including serial passage *in vitro*, DNA damage caused by chemotherapeutic drugs, or the expression of activated oncogenes among others^2^. Recently, programmed cell senescence during embryonic development was shown to contribute to tissue remodeling and morphogenesis of the embryo^3^,^4^, suggesting an evolutionary origin of the process that was co-opted as a useful cell response in the adult^5^. Senescent cells arrest at the G_0_/G_1_ phase of the cell cycle and are characterized by distinctive phenotypic alterations, including enlarged and flattened morphology, enlarged and multinucleated nuclei, and increased senescence-associated beta-galactosidase (SA-beta-gal) activity^6^. In addition, senescent cells secrete a variety of factors to the extracellular environment, collectively known as the senescence-associated secretory phenotype (SASP). This SASP serves to reinforce the senescence arrest in an autocrine manner, but it can also induce growth arrest in a paracrine manner^7^. SASP components are mainly proinflammatory cytokines and chemokines, growth factors, and extracellular matrix remodeling enzymes that can alter the tissue microenvironment and interact with the immune system promoting the recognition and clearance of senescent cells, thereby facilitating the resolution of the damage.

The proinflamatory properties of senescent cells as well as the fact that some viruses have evolved mechanisms to inhibit the senescence process has led to hypothesize that cell senescence may have evolved as an antiviral defense mechanism^8^. The induction of senescence in response to interferon treatment^9^ and the role that endogenous interferon-beta induced by DNA damage^10^ plays in induction of senescence also reinforce this hypothesis.

Here, we explored whether cell senescence plays a role in the host response to viral infection. We found that the replication of vesicular stomatitis virus (VSV) is significantly impaired in senescent cells compared to their non-senescent counterparts. Consistently, we demonstrate that senescence helps to control VSV infection *in vivo*. Finally, we show that the SASP mediates most of the antiviral response and at the same time contributes to activate and recruit the immune system for efficient clearance of the virus. These findings lead us to conclude that cellular senescence protects the organism against virus infection.

## Results

### Replicative senescent mouse fibroblasts are resistant to VSV infection

**Figure 1:**
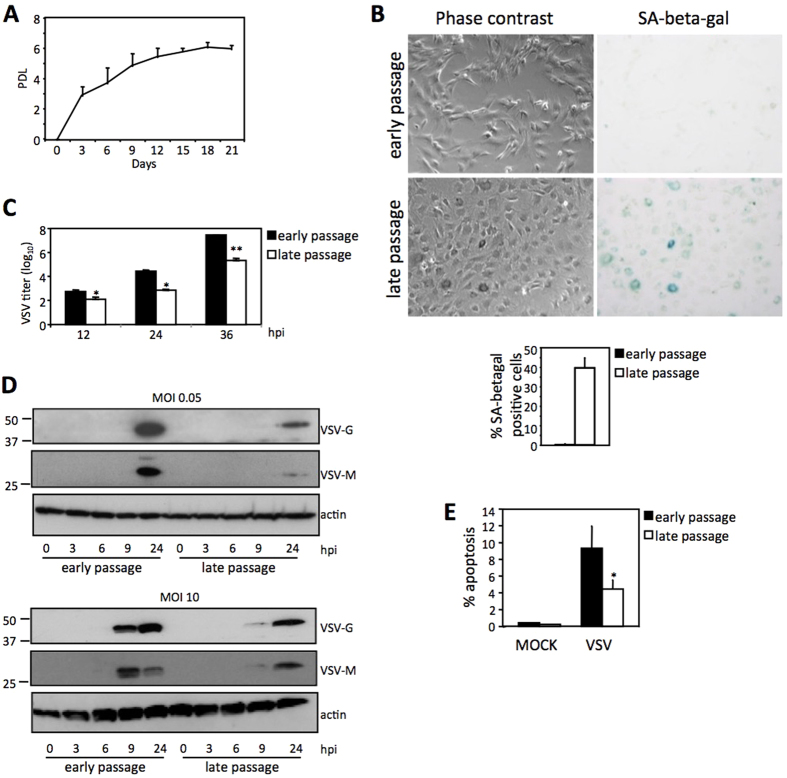
Replicative senescent mouse fibroblasts are resistant to VSV infection. (**A**) Growth curve of serially-passaged MEFs showing accumulated population doublings (PDLs) with time. (**B**) Microscopy images of serially-passaged MEFs showing morphology (left panels) and SA-beta-gal staining (right panels) of early passage (upper panels) and late passage senescent (bottom panels) MEFs. Quantification of the SA-beta-gal positive cells is shown below (at least 200 cells were counted per condition). (**C**) Viral titers (PFU/mL) determined in early or late passage senescent MEFs after the indicated periods of infection (hours post infection, hpi) at a multiplicity of infection (MOI) of 0.05 PFU/cell. (**D**) Western-blot analysis of VSV protein synthesis in early or late passage senescent MEFs after the indicated periods of infection at MOIs of 0.05 PFU/cell (upper panel) or 10 PFU/cell (lower panel). Actin is shown as loading control. (**E**) Percentage of apoptotic cells measured after mock or VSV infection at MOI of 10 PFU/cell, in early or late passage senescent MEFs. Data are mean values +/− SE from at least three different experiments. *p < 0.05, **p < 0.01, ***p < 0.001 Student’s t test.

Cell senescence can result from the activation of different pathways in response to a variety of stressors^2^. We decided to evaluate first whether senescence triggered by a well-established cell-intrinsic mechanism, such as replicative senescence, can function as an antiviral mechanism. Mouse embryo fibroblasts (MEFs) were serially passaged until cell cultures stopped growing and acquired the characteristic senescent morphology showing enlarged and flattened cells (Fig. 1A, B). Then, we analyzed the cells for the expression of the senescence marker senescence-associated beta-galactosidase (SA-beta-gal)^11^. As expected, senescence-arrested MEFs showed increased SA-beta-gal (Fig. 1B).

To test whether replicative senescent cells were more resistant to VSV infection, serially-passaged, or 24 h serum-deprived MEFs were infected with VSV at a multiplicity of infection (MOI) of 0.05 PFU/cell. We quantified VSV particle production at different times after infection by titration of cell supernatants as a measure of virus replication. As shown in Fig. 1C, the titer of virus recovered from replicative senescent MEFs was significantly lower than that recovered from non-senescent MEFs (2.05 × 10^5^ PFU/mL in senescent cells compared to 2.75 × 10^7^ PFU/mL in non-senescent cells, p = 0.008, after 36 h of infection), indicating that replicative senescence limits the replication of VSV in primary mouse cells. This idea was further corroborated by direct inspection of viral protein synthesis after infection of replicative senescent or 24 h serum-deprived MEFs with VSV at a low or a high MOI (0.05 or 10 PFU/cell, respectively) (Fig. 1D). Viral protein synthesis production in the senescent MEFs was clearly lower than in non-senescent MEFs, independently of the MOI tested (Fig. 1D). VSV infection kills infected cells primarily via induction of apoptosis^12^. Thus, we also evaluated the induction of apoptosis triggered after 24 h of infection with VSV at a MOI of 10 in senescent versus non-senescent MEFs. As shown in Fig. 1E, the levels of apoptosis detected in the replicative senescent MEFs after VSV infection were significantly lower than those detected in the non-senescent MEFs (2.03% in senescent cells compared to 8.4% in non-senescent ones, p = 0.048). All together, these results indicated that replication of VSV was limited in replicative senescent MEFs compared to non-senescent cells.

### Chemotherapy-induced senescence of human tumor cells restricts VSV infection

**Figure 2:**
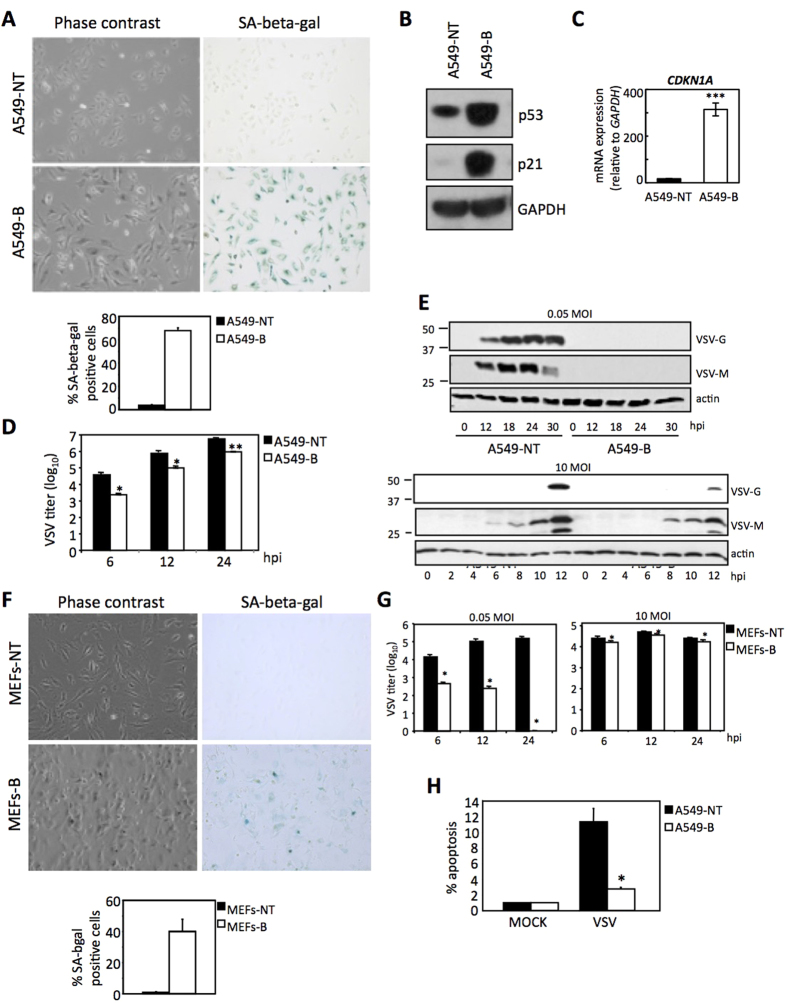
Chemotherapy-induced senescence of human tumor cells restricts VSV infection. (**A**) Microscopy images of human tumor A549 cells showing morphology (left panels) and SA-beta-gal staining (right panels) of untreated (A549-NT, upper panels) and bleomycin-induced senescent (A549-B, bottom panels) A549 cells. Quantification of the SA-beta-gal positive cells is shown below (at least 200 cells were counted per condition). (**B**) Western-blot analysis of senescence markers p53 and p21 in untreated A549 cells (A549-NT) or after bleomycin treatment of A549 cells (A549-B). GAPDH is shown as loading control. (**C**) Expression levels of *CDKN1A* (coding for p21) mRNA relative to *GAPDH* (x10^−3^) as determined by qRT-PCR in untreated (A549-NT) or bleomycin-treated (A549-B) A549 cells. (**D**) Viral titers (PFU/mL) determined in untreated (A549-NT) or bleomycin-treated (A549-B) A549 cells after the indicated periods of infection at a MOI of 0.5 PFU/cell. (**E**) Western-blot analysis of VSV protein synthesis in untreated (A549-NT) or bleomycin-treated (A549-B) A549 cells after the indicated periods of infection at MOIs of 0.05 PFU/cell (upper panel) or 10 PFU/cell (lower panel). Actin is shown as loading control. (**F**) Microscopy images of MEFs showing morphology (left panels) and SA-beta-gal staining (right panels) of untreated (MEFs-NT, upper panels) and bleomycin-induced senescent (MEFs-B, bottom panels) MEFs. Quantification of the SA-beta-gal positive cells is shown below (at least 200 cells were counted per condition). (**G**) Viral titers (PFU/mL) determined in untreated (MEFs-NT) or bleomycin-treated (MEFs-B) MEFs after the indicated periods of infection at MOIs of 0.05 PFU/cell (left panel) or 10 PFU/cell (right panel). (**G**) Percentage of apoptotic cells measured after mock or VSV infection at MOI of 10 PFU/cell, in untreated (A549-NT) or bleomycin-treated (A549-B) A549 cells. Data are mean values +/− SE from at least three different experiments. *p < 0.05, **p < 0.01, ***p < 0.001 Student’s t test.

To test whether the observed resistance to viral infection was a particular feature of mouse cells or specific of senescence-inducing stimulus, we decided to test the viral infection of the human lung adenocarcinoma cell line, A549, rendered senescent by treatment with the chemotherapeutic DNA damaging agent, bleomycin^13^. For this, we first treated A549 cells with bleomycin for 5 days and then we evaluated cells for the presence of cell senescence markers. Bleomycin-treated cells showed increased SA-beta-gal activity and cell size (Fig. 2A), indicative of senescence induction. In addition, we observed elevated expression of the tumor suppressor p53 and of the CDK inhibitor p21Cip1 by Western-blot analysis (Fig. 2B), and of the *CDKN1A* mRNA (the gene coding for p21Cip1) by qRT-PCR (Fig. 2C), indicative of activation of the typical tumor suppressor pathways involved in cell senescence^6^,

Then, bleomycin-induced senescent or 24 h serum-deprived A549 cells were infected with VSV at a MOI of 0.5 PFU/cell, and viral titers in supernatants recovered from infected cells were evaluated at different times after VSV infection. As shown in Fig. 2D, bleomycin treatment induced a statistically significant decrease in viral titers in comparison with untreated cells (9.23 × 10^5^ PFU/mL in senescent A549 versus 4.90 × 10^6^ PFU/mL in control non-senescent A549 cells after 24 h of infection, p-value = 0.006), again indicating that senescence has a role in the control of VSV replication. This notion was further corroborated by direct inspection of viral protein synthesis by Western-blot of cell extracts after infection of bleomycin-induced senescent or 24 h serum-deprived A549 cells, with VSV at low or high MOIs (0.05 and 10 PFU/cell, respectively) (Fig. 2E). While VSV protein synthesis was observed in control cells, viral proteins were virtually undetectable in senescent A549 cells infected with VSV at the low MOI of 0.05 PFU/cell (Fig. 2E, upper panel). At the high MOI of 10 PFU/cell, VSV proteins were detected in senescent A549 cells, but viral protein levels were clearly lower than those observed in the control A549 cells (Fig. 2E, lower panel). Moreover, we also evaluated the effect of bleomycin treatment on the susceptibility of MEFs to VSV replication. We first treated MEFs with bleomycin for 5 days and then we evaluated cells for senescence marker SA-beta-gal activity. As expected, bleomycin-treated MEFs showed increased SA-beta-gal (Fig. 2F), indicative of senescence induction. Bleomycin-treated or 24 h serum deprived MEFs were then analyzed for their viral titers in a similar manner as described above for A549 cells, obtaining similar results (Fig. 2G). To further substantiate our findings, we also evaluated the induction of apoptosis triggered by virus infection in the senescent and control A549 cells, 24 h after infection with VSV at a MOI of 10 PFU/cell. As shown in Fig. 2H, the levels of apoptosis detected in the A549 non-senescent cells after VSV infection were significantly higher than those detected in the senescent A549 cells (11.32% compared to 2.75%, respectively, p = 0.012). These results indicated that senescent A549 cells were significantly more resistant to VSV infection than the non-senescent ones. All together, similarly to what was observed for replicative senescent mouse cells, human tumor cells and mouse primary cells rendered senescent by the DNA damaging agent bleomycin were also less susceptible to VSV infection than non-senescent cells.

### Reduced replication of VSV in oncogene-induced senescent MCF7 cells

**Figure 3:**
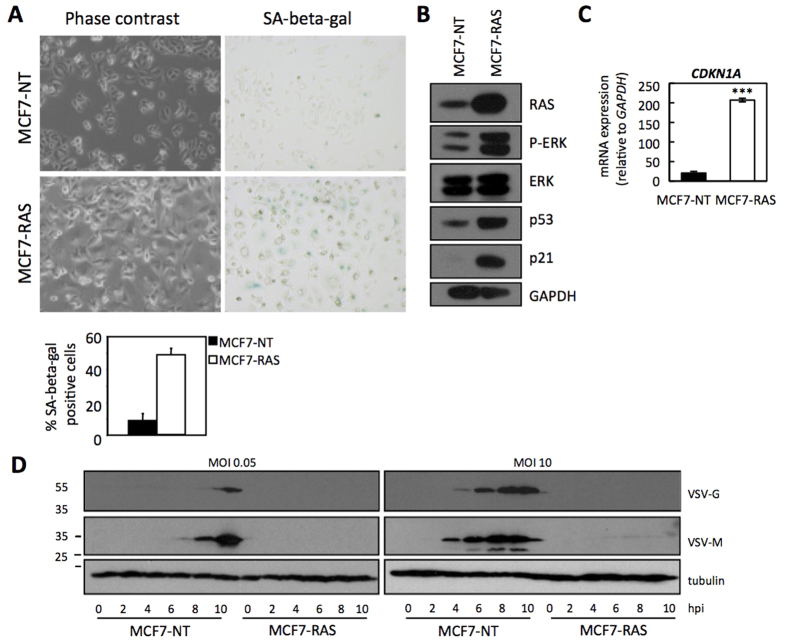
Reduced replication of VSV in oncogene-induced senescent MCF7 cells. (**A**) Microscopy images of human tumor MCF7 cells showing morphology (left panels) and SA-beta-gal staining (right panels) of untreated (MCF7-NT, upper panels) and H-Ras-induced senescent MCF7 cells (MCF7-RAS, bottom panels). Quantification of the SA-beta-gal positive cells is shown below (at least 200 cells were counted per condition). (**B**) Western-blot analysis of H-Ras and downstream targets ERK/P-ERK, and of senescence markers p53 and p21 in untreated (MCF7-NT) or after H-Ras induction (MCF7-RAS) MCF7 cells. GAPDH is shown as loading control. (**C**) Expression levels of *CDKN1A* (coding for p21) mRNA relative to *GAPDH* (x10^−3^) as determined by qRT-PCR in untreated (MCF7-NT) or H-Ras-induced (MCF7-RAS) MCF7 cells. (**D**) Western-blot analysis of VSV protein synthesis in untreated (MCF7-NT) or H-Ras-induced (MCF7-RAS) MCF7 cells after the indicated periods of infection at MOIs of 0.05 PFU/cell (left panel) or 10 PFU/cell (right panel). Tubulin is shown as loading control. Data are mean values +/− SE from at least three different experiments. *p < 0.05, **p < 0.01, ***p < 0.001 Student’s t test.

Our results indicated that intrinsic senescence in primary cells and chemotherapy-induced senescence in primary or tumor cells, function as an antiviral defense mechanism. We then decided to evaluate another well-known senescence-inducing stimulus, in particular oncogene-induced senescence^14^. We first developed an inducible expression system of H-RasV12 in MCF7cells. These cells carry a vector for H-RasV12 that allows inducible expression of the oncogene upon doxycycline addition to the cell culture medium^15^. Addition of doxycycline to MCF7-RAS cells produced a dramatic morphological change that resembles cell senescence (Fig. 3A). SA-beta-gal staining of these cultures showed a clear positive staining when H-Ras was induced compared to the non-treated cells (Fig. 3A). We also verified H-Ras expression and the activation of its downstream MAPK signaling pathway by analysis of the phosphorylated form of ERK by Western-blot after doxycycline treatment (Fig. 3B). In addition, we confirmed the senescence induction by checking the increased levels of p53 and p21 protein (Fig. 3B) and of the CDKN1A mRNA (Fig. 3C). Then, Ras-induced senescent or 24 h serum deprived untreated MCF7 cells were infected with VSV at a MOI of 0.05 or 10 and, at different times after infection, the synthesis of VSV proteins was analyzed by Western-blot. As shown in Fig. 3D, the synthesis of VSV proteins was detected in non-senescent MCF7 cells infected with VSV at a MOI of 0.05 or 10. However, we did not detect VSV protein synthesis in the senescent cells (Fig. 3D), indicating that expression of H-RasV12 induced resistance of MCF7 cells to VSV replication. In summary, these results clearly indicate that senescence induction, independently of the stimulus responsible for triggering the response and in both, human and mouse cells, restricts viral infection.

### Senescence reduces the cell infectivity of VSV

**Figure 4:**
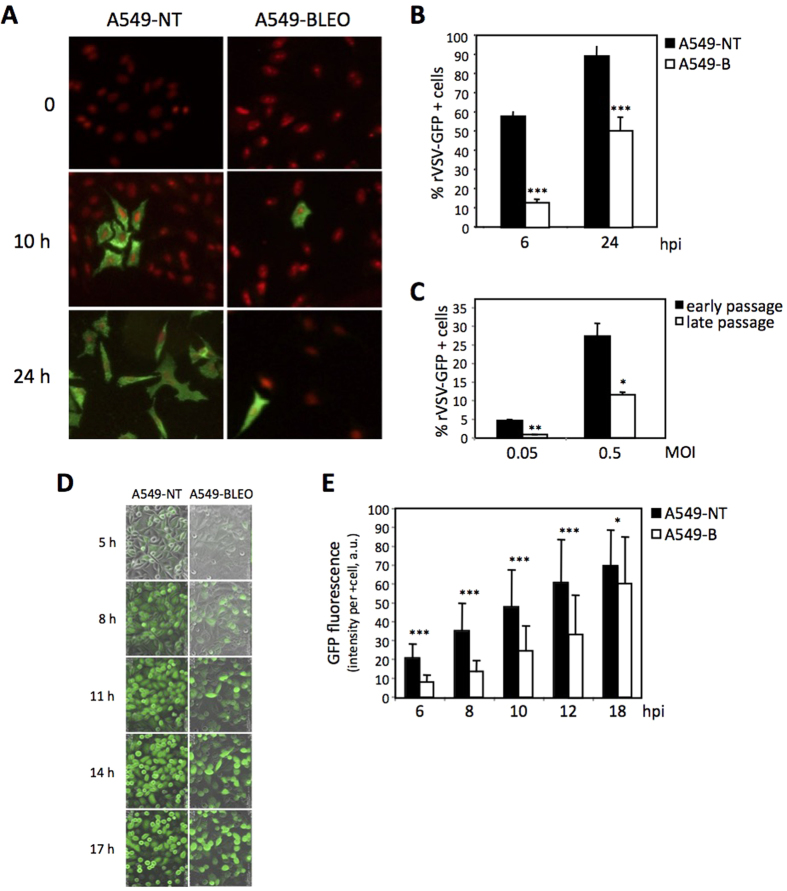
Senescence reduces the cell infectivity of VSV. (**A**) Fluorescent microscopy images of control untreated (A549-NT, left panels) or senescent bleomycin-treated (A549-BLEO, right panels) human tumor A549 cells showing virus spread at different times after infection with recombinant VSV expressing GFP (rVSV-GFP). (**B**) Percentage of rVSV-GFP positive cells after 6 or 24 hours post infection in control untreated (black bars) or senescent bleomycin-treated (white bars) human tumor A549 cells. (**C**) Percentage of rVSV-GFP positive cells after infection with MOIs of 0.05 or 0.5 PFU/cell in control early passage (black bars) or senescent late passage (white bars) MEFs. (**D**) Representative images of control (A549-NT, left panels) or bleomycin-induced senescent (A549-BLEO, right panels) A549 cells infected with VSV-GFP (MOI of 10 PFU/cell) taken at the indicated times. (**E**) Quantification of the intensity of GFP associated fluorescence per GFP positive cell (arbitrary units, a.u.). Data are mean values +/− SE from at least three different experiments. *p < 0.05, **p < 0.01, ***p < 0.001 Student’s t test.

Our results showed that senescence reduces the efficient production of viral particles. Among many possibilities, this could potentially originate from an early event decreasing senescent cell infectivity. To directly assess this possibility, we decided to use A549 cells treated or not with bleomycin, as described above, and exposed to a recombinant VSV expressing GFP (rVSV-GFP). After incubation of the cells with the rVSV-GFP at a low MOI (0.1 PFU/cell), we evaluated the spreading of the virus in the cell culture by immunofluorescence at different times after infection. As shown in Fig. 4A, 24 h after infection virtually all the control non-senescent A549 cells were infected with rVSV-GFP. In contrast, some senescent cells still remained uninfected, proving that senescent cells were less infected by VSV. Then, bleomycin-treated or untreated A549 cells were infected with rVSV-GFP at a higher MOI of 0.5 PFU/cell, and 6 or 24 h after infection we quantified the percentage of rVSV-GFP-positive cells by FACS. Interestingly, we observed a significant reduction in the percentage of GFP-positive senescent cells relative to the GFP-positive non-senescent cells at both times tested (Fig. 4B). Similarly, with a different experimental system, this time using replicative senescent MEFs, we also observed a reduction in the percentage of rVSV-GFP positive senescent cells compared to the non-senescent MEFs at 6 h after infection with VSV at MOIs of 0.05 or 0.5 PFU/cell (Fig. 4C). These results pointed to an early defect in senescent cells that already impairs viral infection. In addition, we also analyzed the evolution of the VSV-GFP infection by video time-lapse microscopy (Fig. 4D) in bleomycin-treated or untreated A549 cells infected at a high MOI (10 PFU/cell). Quantification of the intensity of GFP associated fluorescence, attributable to VSV replication, per infected cell along the experiment revealed that the GFP signal in the infected senescent cells is consistently less intense and evolves more slowly than in the non senescent cells (Fig. 4E). These results clearly establish that senescent cells are not only more resistant to be infected but also less permissive to viral replication.

### The senescence-induced antiviral response is partially mediated by the SASP

**Figure 5:**
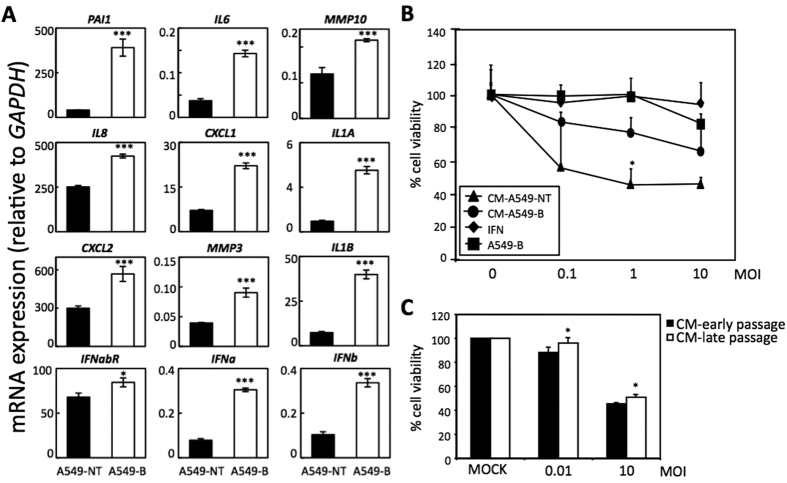
The senescence-induced antiviral response is partially mediated by the SASP. (**A**) Characterization of the expression of different SASP factors by qRT-PCR relative to *GAPDH* (x10^−3^) in control untreated (black bars) or senescent bleomycin-treated (white bars) A549 cells. (**B**) Cell viability of A549 cells cultured with conditioned medium (CM) from control untreated or senescent bleomycin-treated A549, after VSV infection at different MOIs. Viability of senescent bleomycin-treated A549 cells or A549 cells cultured in the presence of interferon are shown as controls. (**C**) Cell viability of MEFs cultures with CM from control early passage or senescent late passage MEFs, after VSV infection at MOIs of 0.01 or 10 PFU/cell relative to mock-infected cells. Data are mean values +/− SE from at least three different experiments. *p < 0.05, **p < 0.01, ***p < 0.001 Student’s t test.

One of the properties of the senescent cells is the secretory phenotype^7^. Senescent cells secrete a variety of proteins including cytokines, chemokines, extracellular matrix remodeling enzymes, and growth factors that modify the tissue microenvironment. Some of these factors have a known role in the innate immune response^16^. Therefore, we hypothesized that the senescence-induced antiviral response could be potentially mediated by the SASP. To evaluate this hypothesis, we examined the effect of senescent conditioned media (CM) on VSV replication. We first characterized the SASP produced by A549 cells induced to senesce by bleomycin treatment. The expression of different SASP components, including some interferon genes (*IFNa*, *IFNb*, and *IFNabR*), was measured by qRT-PCR, confirming the increased levels of several of these factors (Fig. 5A). Then, A549 cells were incubated with CM from bleomycin-induced senescent or control A549 cells for 24 h, and cells were then infected with VSV at different MOIs. Evaluation of the cytopathic effect 24 h after infection revealed that cells incubated with the senescent CM showed a significantly higher percentage of viable cells than those incubated with control CM from non-senescent cells at a MOI of 1 PFU/cell (Fig. 5B) (77.01% in cells cultured with senescent CM compared to 56.59% when cultured in control CM, p = 0.008). This increased protection conferred by the senescent medium was not as high as the protection observed directly using bleomycin-induced senescent A549 cells or when interferon was added to the medium as a control. This indicates a higher resistance of cells to VSV infection when cultured in the presence of senescent CM. Similarly, we also observed that incubation of MEFs with CM from replicative senescent cells increased viability 24 h after infection with VSV (MOI of 0.01 or 10 PFU/cell) (Fig. 5C). All together, these results suggest that the SASP has at least a partial role in the control of virus infection mediated by cell senescence.

### Senescence induction *in vivo* restricts viral infection in mice

**Figure 6:**
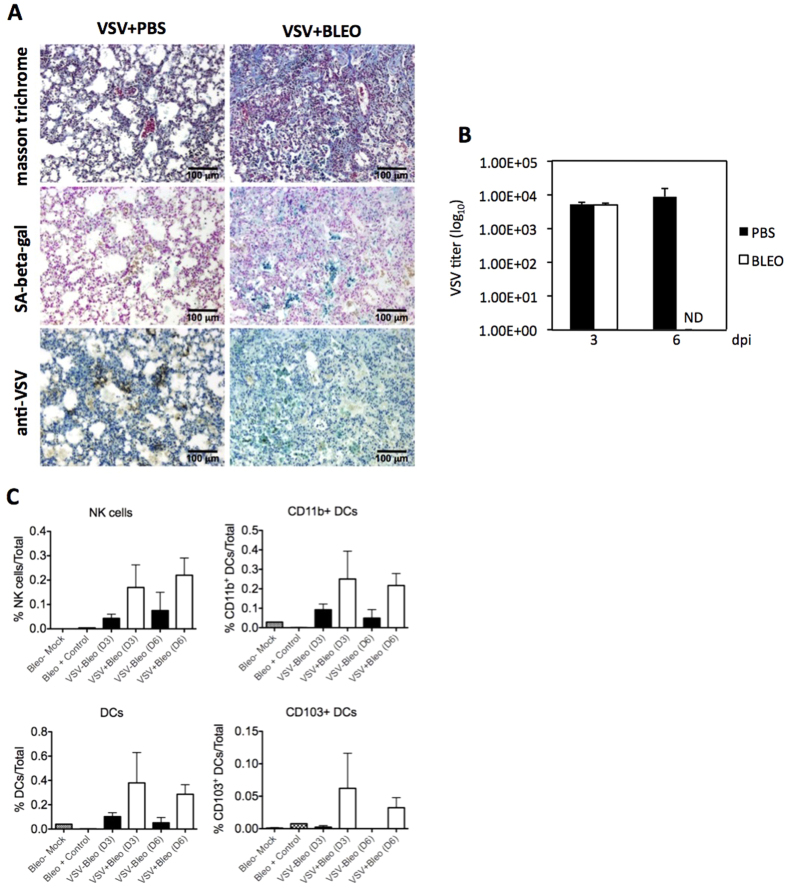
Senescence induction *in vivo* restricts viral infection in mice. (**A**) Lung sections from mice intratracheally treated with PBS (left panels) as control or bleomycin (right panels) to induce senescence, stained with masson trichrome (upper panels), SA-beta-gal (middle panels), of IHC against VSV-G (lower panels). (**B**) Viral titers (PFU/gr) determined from lungs of mice intratracheally treated with PBS (black bars) as control or bleomycin (white bars) to induce senescence, after 3 or 6 days of intranasal administration of VSV. (**C**) Characterization of immune cell populations in lungs from mice intratracheally treated with PBS (black bars) as control or bleomycin (white bars) to induce senescence, after 3 or 6 days of intranasal administration of VSV.

Our findings clearly indicated that replication of VSV was significantly impaired in senescent cells in culture. To substantiate these observations, we decided to evaluate the putative antiviral activity of cell senescence *in vivo*. For this, bleomycin or PBS control was administered intratracheally to mice in order to induce lung fibrosis and cell senescence as previously described^13^. Twenty one days after treatment, mice were infected intranasally with VSV (1 × 10^5^ PFU/mice), and at 3 or 6 days after infection mice were sacrificed, and lungs were removed to determine viral titers. First, we evaluated the presence of senescent cells in the lungs of treated mice by performing whole-mount SA-beta-gal staining. Stained lungs were embedded in paraffin, sectioned and further stained to reveal fibrosis using masson trichrome staining, and to detect VSV particles by immunohistochemistry (IHC). As shown in Fig. 6A, lungs from bleomycin-treated mice presented clear signs of fibrosis as judged by masson trichrome staining, as expected. At the same time, the fibrotic bleomycin-treated lungs showed a strong positive SA-beta-gal staining in consecutive sections, in comparison with the negative staining of lungs from control PBS-treated mice (Fig. 6A). We then determine the viral titers recovered from lung extracts and showed that, at 3 days post-infection, similar VSV titers were detected in the lungs of mice independently of the administration of bleomycin or PBS (Fig. 6B). Interestingly however, we observed a clear difference in the viral titers measured in the control group at 6 days after infection compared to the senescence group (Fig. 6B). In particular, we could not detect VSV in any of the bleomycin-treated mice at 6 days after infection, while control mice still continued producing high titers of virus. These results were corroborated by IHC against VSV in lung sections stained in parallel with masson trichrome and SA-beta-gal. As shown in Fig. 6A, at 6 days post-infection, lungs from bleomycin-treated mice show very weak, if any, signal of viral infection, while the control tissue was clearly positive by IHC for VSV (22.89% positive cells in control versus 2.5% in bleomycin-treated lungs). These results indicated that cell senescence could function as an efficient mechanism to control virus replication *in vivo*.

Previous studies have indicated that SASP production leads to clearance of senescent cells via recruitment of innate immune cells^17^,^18^. Thus, we reasoned that the viral clearance observed in bleomycin-treated mice at day 6 could be dependent, at least to some extent, on the removal of infected cells by infiltrating immune cells. To test this hypothesis, we utilized multiparametric flow cytometry to determine the phenotype of cells in the bronchoalveolar lavage (BAL) of treated and control mice after VSV infection. Our results indicated that both NK cells as well as conventional DCs (CD11b^+^ and CD103^+^) were significantly upregulated in the BAL of VSV-infected mice treated with bleomycin (Fig. 6C). While these findings do not imply causality, they strongly suggest that mechanistically, the SASP influence the recruitment of immune cells with capacity to clear virus from the sites of primary infection.

## Discussion

Cellular senescence had a widely recognized status as a stress response mechanism triggered to prevent excessive proliferation of damaged cells caused either by accumulation of rounds of cell division or by hyperproliferative signals emanating from mutated oncogenes within the cell^2^. This concept was expanded to include also external signals such as chemotherapeutic drugs or wound healing. The recent discovery of programmed cell senescence operating during embryo development opened even further our view of cell senescence^3^,^4^. In this context, cell senescence operates as a morphogenic force that relies on secreted factors to recruit immune cells to clear out unneeded cells to allow for cell replacement, among other possible functions^5^. This basic mechanism might have been retained by evolution to operate during adulthood in settings such as cancer prevention or tissue repair. The stable cell cycle arrest and the release of proinflammatory cytokines and chemokines that characterizes cell senescence may evoke features of antiviral responses. Reinforcing this idea, numerous viruses have evolved mechanisms to interfere with senescence, a fact that might be interpreted as a viral strategy to escape from the cellular antiviral system^8^. Here, we wanted to explore the antiviral power of cell senescence, using VSV as a model and testing different senescence stimuli in primary or tumor cells, of mouse or human origin.

Our observations clearly establish that, independently of the trigger, senescence reduces the replication of VSV. This translates into low viral titers recovered from supernatants of infected cells, reduced viral protein synthesis, or decreased apoptosis after infection in senescent cells. Furthermore, we observed that senescence restricts the infectivity of VSV. Incubation with conditioned medium produced by senescent cells is enough to confer protection to proliferating cells against virus infection, indicating that this antiviral response is at least in part mediated by a complex secretory response that characterizes cell senescence, and known as the SASP. This senescence-mediated antiviral activity was validated *in vivo* when control or bleomycin-treated mice were challenged with VSV. Importantly, lungs from *in vivo* bleomycin-induced senescence mice showed no signs of viral particles after 6 days of infection compared to the high levels of virus detected in control mice. This suggests that the senescence derived SASP could be participating in the activation and recruitment of immune cells to the site of infection. A similar role of SASP promoting clearance of damaged cells from tissues and allowing for repair has been previously described for cell senescence in cancer or wound healing settings^5^. This new function of cell senescence as an antiviral mechanism provides new links between antitumor and antiviral immunity.

## Materials and Methods

### Cells, virus, reagents and mice

Wild type mouse embryo fibroblasts (WT MEFs) were extracted from 13.5 days embryos and cultured in complete medium (Dulbecco’s Modified Eagle’s Medium (DMEM, Sigma) supplemented with 10% heat inactivated foetal bovine serum (FBS, Sigma), 1% penicillin/streptomycin (10.000 U penicillin/10 mg streptomycin, Sigma) and 2 mM L-glutamine (200 mM, Sigma), at 37 °C and in 20% oxygen and 5% CO_2_. Cells were serially passaged until they entered senescence. BSC-40, A549, and MCF7 cells were maintained in complete medium. Infections were carried out using VSV of Indiana strain or recombinant VSV expressing GFP (rVSV-GFP) kindly provided by Dr. Adolfo Garcia-Sastre (Mount Sinai School of Medicine), and virus yields were measured by plaque assays in BSC-40 cells. Cells were treated with 50 μg/ml bleomycin (15 UI, Nippon Kayaku) for 5 days to induce senescence. Mice (C57BL/6) were housed at the serum pathogen-free (SPF) barrier area of CIMUS-USC. All experiments were carried out in compliance with Principles of Laboratory Animal Care of national laws, and all animal procedures were performed according to protocols approved by the Santiago de Compostela University Bioethics Committee (protocol number 15005AE/07/01/02/05 C/AVF2).

### SA β-gal and masson trichrome stainings

Senescence-associated β-galactosidase (SA-beta-gal) activity was determined as reported previously^11^. Whole-mount SA-beta-gal staining was performed as previously described^3^. After staining, tissue was embedded in paraffin and sectioned for masson trichrome staining or immunohistochemistry with antibody against VSV-G. Photographs were taken in an Axio Vert.A1 microscope (Zeiss) with ZEN software.

### Antibodies

Anti-VSV M antibody was from KeraFAST, anti-p53 antibody (DO-1) and anti-p21 (F-5) were from Santa Cruz (#sc126 and sc-6246, respectively), anti-P-Erk (P-p44/42) and anti-Erk (p44/42) were both from Cell Signalling Technologies (#4370 and #4695, respectively), anti-Pan-Ras (OP40) was from Millipore, anti-actin was from MP Biomedicals, and anti-tubulin antibody was from Serotec. VSV-G antibody was a generous gift of Dr. I Ventoso (CBMSO, Madrid).

### Protein and RNA expression analysis

For protein expression analysis, cell extracts were prepared using RIPA buffer (150 mM NaCl, 10 mM Tris-HCl pH 7.5, 0.1% SDS, 1% Triton X100, 5 mM EDTA pH 8.0, 1% Deoxycholate and sodium salt containing protease inhibitors), and appropriate volumes of cell extracts, adjusted to represent the same amount of total cellular protein (30 μg), were electrophoresed in 12% polyacrylamide gels. After electrophoretic transfer to a PVDF membrane at 100 V for 1 h at 4 °C, the membrane was blocked with 5% milk in TTBS (20 mM Tris-HCl pH7.5, 150 mM NaCl, 0.05% Tween-20) for 1 h at room temperature. Membranes were incubated at 4 °C overnight with primary antibodies as indicated. Incubation with the appropriate secondary antibodies conjugated to HRP was followed by visualization using the ECL system.

To measure RNA expression, total RNA was extracted using the NucleoSpin^®^ RNA kit (Macherey-Nagel) following the indications of the provider and DNAse treatment. After nanodrop RNA quantification, the RNA was retrotranscribed into cDNA according to the manufacturer’s protocol (High-Capacity cDNA Reverse Transcription Kit, Applied Biosystems). Quantitative Real Time-PCR was performed using SYBR Green Power PCR Master Mix (Applied Biosystems) in an Mx3005 P real-time PCR system (Agilent technologies Stratagene). Relative quantitative RNA was normalized using the housekeeping gene *GADPH.* Primer sequences are available from the authors upon request.

### Immunofluorescence and time-lapse microscopy

Immunofluorescence staining was performed as previously described^19^. Analysis of the samples was carried out on an Axio Vert.A1 microscope (Zeiss) with ZEN software. For time-lapse microscopy, A549 cells grown on culture-insert microwells (80406, Ibidi) were infected with VSV-GFP at a MOI of 10 PFU/cell. Pictures were taken every 10 min starting at 5 hours post infection (hpi) and until 18 hpi on a Nikon BioStation microscope using BioStation IM software (v2.12, build 136, Nikon). We quantified the intensity of the GFP associated fluorescente per infected cell using NIS-Elements BR (v.4.13, Nikon).

### Flow cytometry

Cells infected with rVSV-GFP were fixed in 2% paraformaldehyde for 20 min. The percent of GFP positive cells was determined by flow cytometry, using a FACScalibur cytometer (BD Biosciences).

### Apoptosis quantification

Apoptosis was quantified by flow cytometry using the caspase-3, active form, mAb Apoptosis kit from BD Pharmigen, according to manufacturer’s protocol.

### Effect of conditioned media on VSV replication

A549 cells were forced into senescence by cultivation with bleomycin for 5 days. The cultivation medium was then removed and the cells were cultivated in fresh drug-free 2% FBS medium for the following 3 days. The conditioned medium produced by senescent cells was filtered through PVDF syringe driven filters (0.22 μm, Jet-Biofil) and transferred to normal proliferating A549 cells. Control cells were treated in the same manner by conditioned medium recovered from normal A549 cells. After 24 h of cultivation in conditioned medium, cells seeded into 96-well plates were infected with VSV at different MOIs. After 24 h of infection, the medium was removed and cytolysis was determined by crystal violet staining as described previously^20^. The percentage of viable cells was calculated assuming the survival rate of uninfected cells to be 100%. Conditioned medium from replicative senescent MEFs or from proliferating MEFs was assayed in a similar manner. A549 cells or MEFs treated with 500 U/mL of human or mouse β-interferon, respectively, were included as control.

### *In vivo* analysis of viral infection

Eight weeks old littermate male C57BL/6 mice were used in this study. To induce pulmonary fibrosis, bleomycin was administered intratracheally at a dose of 5 mg/Kg body weight (n = 7 mice). A control group (n = 13 mice) received the same volume of sterile saline only. At 21 after treatment, 6 mice per group were intranasally infected with a single dose of VSV (10^5^ PFU), and at 3 or 6 days after infection, mice were sacrificed and their lungs removed. In addition, we collected bronchoalveolar lavages (BAL) for immune cell population analysis. A fraction of the lungs was used for histological analysis as mentioned above, and the rest was immediately frozen for viral titer determination.

### Statistical analysis

For statistical analysis between control and different groups the Student’s t test was applied. The significance level chosen for the statistical analysis was p < 0.05.

## Additional Information

**How to cite this article**: Baz-Martínez, M. *et al.* Cell senescence is an antiviral defense mechanism. *Sci. Rep.* **6**, 37007; doi: 10.1038/srep37007 (2016).

**Publisher’s note:** Springer Nature remains neutral with regard to jurisdictional claims in published maps and institutional affiliations.

## References

[b1] Hayflick L. & Moorhead P. S. The serial cultivation of human diploid cell strains. Exp Cell Res 25, 585–621 (1961). 13905658 10.1016/0014-4827(61)90192-6

[b2] Collado M., Blasco M. A. & Serrano M. Cellular senescence in cancer and aging. Cell 130, 223–233 (2007). 17662938 10.1016/j.cell.2007.07.003

[b3] Munoz-Espin D. *et al.* Programmed cell senescence during mammalian embryonic development. Cell 155, 1104–1118 (2013). 24238962 10.1016/j.cell.2013.10.019

[b4] Storer M. *et al.* Senescence is a developmental mechanism that contributes to embryonic growth and patterning. Cell 155, 1119–1130 (2013). 24238961 10.1016/j.cell.2013.10.041

[b5] Munoz-Espin D. & Serrano M. Cellular senescence: from physiology to pathology. Nat Rev Mol Cell Biol 15, 482–496 (2013). 10.1038/nrm3823 24954210

[b6] Collado M. & Serrano M. The power and the promise of oncogene-induced senescence markers. Nat Rev Cancer 6, 472–476 (2006). 16723993 10.1038/nrc1884

[b7] Perez-Mancera P. A., Young A. R. & Narita M. Inside and out: the activities of senescence in cancer. Nat Rev Cancer 14, 547–558 (2014). 25030953 10.1038/nrc3773

[b8] Reddel R. R. Senescence: an antiviral defense that is tumor suppressive? Carcinogenesis 31, 19–26 (2010). 19887513 10.1093/carcin/bgp274

[b9] Moiseeva O., Mallette F. A., Mukhopadhyay U. K., Moores A. & Ferbeyre G. DNA damage signaling and p53-dependent senescence after prolonged beta-interferon stimulation. Mol Biol Cell 17, 1583–1592 (2006). 16436515 10.1091/mbc.E05-09-0858 PMC1415317

[b10] Yu Q. *et al.* DNA-damage-induced type I interferon promotes senescence and inhibits stem cell function. Cell Rep 11, 785–797 (2015). 25921537 10.1016/j.celrep.2015.03.069 PMC4426031

[b11] Dimri G. P. *et al.* A biomarker that identifies senescent human cells in culture and in aging skin *in vivo*. Proc Natl Acad Sci USA 92, 9363–9367 (1995). 7568133 10.1073/pnas.92.20.9363 PMC40985

[b12] Balachandran S., Porosnicu M. & Barber G. N. Oncolytic activity of vesicular stomatitis virus is effective against tumors exhibiting aberrant p53, Ras, or myc function and involves the induction of apoptosis. J Virol 75, 3474–3479 (2001). 11238874 10.1128/JVI.75.7.3474-3479.2001 PMC114141

[b13] Aoshiba K., Tsuji T. & Nagai A. Bleomycin induces cellular senescence in alveolar epithelial cells. Eur Respir J 22, 436–443 (2003). 14516132 10.1183/09031936.03.00011903

[b14] Serrano M., Lin A. W., McCurrach M. E., Beach D. & Lowe S. W. Oncogenic ras provokes premature cell senescence associated with accumulation of p53 and p16INK4a. Cell 88, 593–602 (1997). 9054499 10.1016/s0092-8674(00)81902-9

[b15] Alexander E. *et al.* IkappaBzeta is a regulator of the senescence-associated secretory phenotype in DNA damage- and oncogene-induced senescence. J Cell Sci 126, 3738–3745 (2013). 23781024 10.1242/jcs.128835

[b16] Acosta J. C. *et al.* A complex secretory program orchestrated by the inflammasome controls paracrine senescence. Nat Cell Biol 15, 978–990 (2014). 10.1038/ncb2784 PMC3732483 23770676

[b17] Lujambio A. To clear, or not to clear (senescent cells)? That is the question. Bioessays 38 Suppl 1, S56–S64 (2016). 27417123 10.1002/bies.201670910

[b18] Sagiv A. & Krizhanovsky V. Immunosurveillance of senescent cells: the bright side of the senescence program. Biogerontology 14, 617–628 (2013). 24114507 10.1007/s10522-013-9473-0

[b19] Campagna M. *et al.* Rotavirus viroplasm proteins interact with the cellular SUMOylation system: implications for viroplasm-like structure formation. J Virol 87, 807–817 (2013). 23115286 10.1128/JVI.01578-12 PMC3554093

[b20] Garcia M. A. *et al.* Antiviral action of the tumor suppressor ARF. Embo J 25, 4284–4292 (2006). 16957780 10.1038/sj.emboj.7601302 PMC1570439

